# The New and Key Roles for Psychological Contract Status and Engagement in Predicting Various Performance Behaviors of Nurses

**DOI:** 10.3390/ijerph192113931

**Published:** 2022-10-26

**Authors:** John Rodwell, Andre Gulyas, Dianne Johnson

**Affiliations:** 1 Department of Management & Marketing, Swinburne University of Technology, Hawthorn, VIC 3122, Australia; 2 Think HQ, South Melbourne, VIC 3205, Australia; 3 Griffith Business School, Griffith University, Brisbane, QLD 4111, Australia

**Keywords:** psychological contract, nurses, engagement, organizational citizenship behavior, job satisfaction, performance

## Abstract

The study examines the impact of the psychological contract (PC), including the often-studied PC breach in addition to the novel approach of PC status, as predictors of performance among nurses, mediated by engagement, job satisfaction, and psychological distress. A sample of 177 nurses and midwives from a medium to a large hospital in Australia completed a self-report questionnaire. Structural equation modelling was used to determine associations between the predictors (i.e., negative affectivity (NA), PC breach, PC status)), mediating variables (i.e., engagement, job satisfaction, and psychological distress), and three types of performance behaviors: organizational citizenship behavior for the individual, for the organization (OCBI, OCBO) and in-role behavior (IRB) simultaneously. Specifically, psychological contract status positively predicted engagement, whereas breach negatively predicted engagement and positively predicted job satisfaction. NA positively predicted distress, and distress negatively predicted OCBO and IRB. Lastly, engagement positively predicted job satisfaction, OCBI, OCBO, and IRB. The findings indicate that psychological contract status may predict engagement (and in turn, performance) over and above psychological contract breach, and thus this novel construct should be examined further. The importance of engagement for predicting the performance behaviors and mental health of nurses may also offer new insights.

## 1. Introduction

Nurses make up the largest group of professionals in healthcare organizations [1] and working on the frontline means that their performance is vital for patient satisfaction and patient outcomes [2,3]. Moreover, the ongoing nursing shortage means that in addition to in-role responsibilities, any extra effort nurses put into their work beyond their formal roles can be important to maintaining their services [4]. Fortunately, the psychological contract (PC) may represent an ideal way to maintain high IRB and OCB in relation to the fulfilment of promises. That is, organizations’ failures to adequately fulfil promises may result in poor outcomes (e.g., job dissatisfaction) among nurses [5,6], which may lead to decreased performance [7,8,9], and a higher incidence of burnout [10,11].

The PC is an aspect of the employee-employer social exchange relationship, whereby employees perceive promises and obligations made by the employer in exchange for their own efforts [12]. Examining the PC among nurses allows researchers to investigate the quality of nurses’ relationships with their employers that may enable improvements to their performance reflected in both IRB and OCB. For example, enabling employees to respond positively to stressful work situations [13]. In addition to a potential direct impact of PC on performance, previous findings illustrate the importance of the PC in predicting several employee outcomes that may precede performance, such as work engagement, job satisfaction, and mental health [6,14,15,16,17], along with increased engagement with healthy behaviors [18]. Although engagement appears to be an important outcome in exchange relationships [19] and there are calls to include aspects of psychological health in PC studies [20], few studies explore these outcomes with the PC.

In addition to exploring the aforementioned outcomes and performance with the PC, multiple aspects of the PC can be examined. For instance, PC breach has been the main focus of much of PC research, although the direct effects of a breach on employees are inconsistent and often have weak effect sizes [21]. The current paper introduces a new way of capturing the breadth of the PC, including under-, just-, and over-fulfilment of the PC, known as PC status. PC status refers to an overall ‘tally’ of aspects of PC fulfilment and provides a more holistic view of the employment relationship over time than PC breach, capturing the positive, negative,, and neutral aspects of the PC rather than solely reflecting negative events such as PC breach.

Previous findings illustrate potential relationships between the PC and employee performance with potential mediators, such as engagement, job satisfaction, and mental well-being [11,13]. However, studies need to consider all of these interconnected variables simultaneously. Individual differences in personality may also influence the relationship between the PC and its outcomes. Specifically, negative affectivity (NA) may increase sensitivity to PC breaches and status [22]. Therefore, the current study aims to investigate the relationship between two indicators of the condition of the employee-employer exchange relationship (PC status and PC breach) and employee-level performance, as mediated by engagement, job satisfaction, and psychological distress, while accounting for NA’s potential influence on these relationships, among nurses.

### 1.1. Social Exchange Theory and Aspects of the Psychological Contract

Social exchange theory (SET) relates to how a give-and-take relationship between two parties evolves over time. These two-way relationships are mutually beneficial where each party is expected to act to benefit the other party and expects those efforts to be reciprocated; if efforts are over or under-reciprocated by one party then the other party may adjust their inputs accordingly [23].

The psychological contract (PC) representing a specific form of a social exchange relationship between an employee and their employer, was first described decades ago (e.g., [24]) and has more recently gained popularity through the work of Rousseau and colleagues, who further defined the concept [12,25]. The PC entails the perceived written and unwritten agreements between an employee and their employer, which are based on employee expectations of their employer stemming from perceived promises. These obligations can include but are not limited to, promotions, pay for performance, and/or training provided by the employer [12].

Alternatively, PC fulfilment can also be thought of as reflecting the degree of socio-emotional and economic resources provided to the employee, such as any psychological, physical, social, and organizational resources that help an employee do their work effectively [19,26]. Therefore, ongoing fulfilment of the PC may motivate and enable employees to perform well and increase their performance due to feeling a need to reciprocate what they receive from the organization while expecting that their efforts will continue to be reciprocated in the future.

In contrast, a PC breach represents one or more events where promises regarding the employees’ psychological contracts are perceived to be broken and may have detrimental effects on employees [27]. According to SET and the norm of reciprocity, a breach in the PC would be expected to lead to employees decreasing their performance or withdrawing their engagement to balance the social exchange relationship with their organization [28]. Further, perceived PC breach also means that the employee feels that they are not receiving what they want from their job [6], thus they are likely to have low job satisfaction as a result.

A key consequence of a PC breach is likely to be an affective, rather than a cognitive, response [6,29,30], whereby a breach may lead to feelings of anxiety, betrayal, and violation, and these feelings may reflect an increase in psychological distress [27]. That is, a PC breach may influence employees’ emotions and potentially destroy their perceptions of the reciprocal nature of the employee-employer relationship [31]. PC breach has been identified as an important antecedent of low engagement [16], low job satisfaction [17], poor mental well-being [32], and low IRB and general OCB [33,34].

#### 1.1.1. Introducing PC Status

Although PC breach has been the main focus of PC studies, the direct effects of breaches on employees have been inconsistent with weak effect sizes [21], and therefore the perspective that PC breach offers may be too narrow by itself. A new and relatively novel approach to the PC, referred to as PC status, provides a different perspective of the PC and may provide insight that PC breach and fulfilment do not.

The norm of reciprocity that underpins social exchange theory explains the positive associations between PC fulfilment and indicators of employee performance [23]. The norm of reciprocity surmises that successful fulfilment of the PC from both the employee and their employer encourages the employee to continue to reciprocate by increasing their performance, more easily through OCB but also IRB, while also reinforcing expectations that their employer will continue to fulfil, or over-fulfil, the PC [23,35].

PC status considers obligations and fulfilment together and is characterized by an employee’s overall perception of whether their employer is over-, just-, or under-fulfilling the PC while considering the degree of importance of each obligation to the employee by weighting each obligation accordingly. Weighting the variable in this manner leads to a more comprehensive view of the general status of the PC for each employee. For example, when the PC is over-fulfilled there may be a trade-off between over-fulfilment and under-fulfilment of specific perceived obligations. That is, in cases where some areas of the PC are over-fulfilled, the employee may be less inclined to perceive under-fulfilment of the PC overall [22], which would be represented by high PC status. It may also be that if an important obligation in the PC is under-fulfilled, over-fulfilment of other, less important obligations may not prevent the negative impact of a poor PC state.

Despite some researchers having examined fulfilment weighted by importance (e.g., [36]), to the best of the authors’ knowledge, the gap between obligations and fulfilment has yet to be assessed in this way. PC status may be positively related to employees’ expectations of future reciprocation by their employer and these expectations may be the mechanism that motivates employees to engage in their work. High PC status may encourage and enable an employee to act in ways that benefit the organization and others within it. That is, high PC status that is built up over time may reflect employees’ strong belief that their employer will satisfy the PC based on previous successful exchanges, which may lead to increased motivation reflected in work engagement which may subsequently enable more resources and increase task performance and OCB.

PC status may provide a more informative and comprehensive representation of the PC than PC fulfilment or PC breach alone. Some researchers have examined PC fulfilment and PC breach weighted by the importance of each obligation (e.g., [22,36]), however, they have yet to investigate the discrepancy between obligations and fulfilment weighted by importance as represented by PC status. Consequently, PC status provides a novel way of examining the PC whereby high PC status would reflect successful ongoing mutual reciprocation of the PC across the employee-employer exchange relationship.

#### 1.1.2. Distinctiveness of PC Breach and PC Status

Some researchers may argue that PC breach and PC status are essentially the same constructs and therefore only one should be examined. However, although PC breach, PC fulfilment, and PC status initially appear to be similar concepts along a continuum, with many researchers using the terms interchangeably [6,29], there are calls to treat them as distinct concepts [30,37]. There are several indications that fulfilment as a component of PC status and breach relates to different aspects of the PC, with each providing unique insights. First, PC status does not indicate whether a PC breach has been perceived. Second, PC breach and fulfilment, and by extension PC status, are qualitatively different, as demonstrated by the differences between how they are each measured. Measures of fulfilment normally ask participants to indicate the extent that specific obligations have been fulfilled (e.g., [19]), whereas breach is more broadly measured, asking participants the extent that their organization has broken the PC in general (e.g., [32]). Third, PC status and a breach may impact different outcomes through different mechanisms. For example, the status may have greater effects than breach on employees, whereby what employees receive from their employer may be of greater importance than the gap between their expectations or the employer’s promises and what they actually give [30]. Conversely, other evidence suggests that PC breaches may have a larger impact than fulfilment or status on employees, possibly because negative events are more impactful than positive outcomes on individuals’ moods [38]. A PC breach is also likely to damage employees’ perceptions of the likelihood of reciprocation in their PC, yet low PC status may not. Ultimately, PC breach and PC status (reflecting weighted PC fulfilment) should be considered together as they are likely to uniquely impact employee outcomes [38].

Though there is evidence for the direct effects of the PC on performance, there may be variables that mediate this relationship, as indicated by inconsistencies and weak effect sizes of the PC directly onto employee outcomes [21]. The PC positively relates to work attitudes such as engagement, job satisfaction, and mental health [19,39], among other outcomes (such as perceived insider status in an organization [10]), and that these variables further relate to performance (e.g., [7,9,40]) and thus may at least partially mediate the relationship between aspects of the PC and types of employee performance.

#### 1.1.3. The Potential Mediators of Engagement, Job Satisfaction and Psychological Distress

Work engagement has been found to play a strong role in predicting general work performance [14]. Engagement has been positively linked to IRB and OCB in separate studies [8,40,41], however, IRB and OCB are rarely included simultaneously in studies [42]. Engagement may significantly relate to OCBI but not OCBO [43], although the participants were shop floor employees in the manufacturing industry in Sri Lanka so the findings may not generalize to other professions, cultures, or locations such as nurses in Australia.

The norm of reciprocity and SET may provide the mechanisms underlying the impact of PC on engagement and engagement’s impact on performance. From a norm of reciprocity perspective, increasing engagement could also be seen as a way for employees to reciprocate the organizations’ efforts, whereas employees may lower engagement as a way of withdrawing from their work in response to the organization’s failure to meet their expectations [23].

Further, if employees with high levels of engagement tend to exhibit more OCB and IRB, the relationship between the PC and performance may be partially mediated by engagement. Specifically, high PC status may instill a sense in the employee of owing the organization and the employee may reciprocate an over-fulfilment of the PC to balance the exchange relationship, potentially by increasing their own engagement (reflecting increased motivation and personal energy invested into work). Increasing engagement to perform better may require the employee to gather more resources to enable them to put more energy into their work. Therefore, the organization’s over-fulfilment of the PC may increase employees’ OCB and IRB either by compelling the employee to reciprocate with in-role performance or going beyond what is formally required to balance the exchange relationship [44].

Alternatively, the PC may also act as resources (e.g., cognitive, emotional, and physical) that are required to engage in work, and therefore ‘paying off’ the exchange relationship by increasing engagement may require employees to have sufficient resources of their own [8,19]. Employees with more resources over time are more likely to have higher engagement with their work and are also more likely to build more resources and seek opportunities where they are better able to engage [45].

In addition to employee engagement, job satisfaction and psychological distress may also play mediating roles between the PC and employee performance, with evidence suggesting that they may be important predictors of employee performance [7,9,14,15] and may also be predicted by the PC [30,37,39]. Specifically, job satisfaction can be conceptualized as attitudes reflecting evaluations of the job situation by the employee regarding what they want from a job and what they get, which are conceptually similar to aspects of the PC and therefore job satisfaction may reflect the employees’ level of satisfaction with the PC [6]. Moreover, a high degree of PC fulfilment or low PC breach has been found to positively relate to job satisfaction [6,37,39], and job satisfaction also appears to positively predict OCB [15] including both OCBI and OCBO [7].

Conversely, a poor PC may decrease an employees’ mental wellbeing [32], although because of its stronger potential to increase negative emotions PC breach may influence mental wellbeing more strongly than PC fulfilment [38], which in turn would be expected to decrease an individual’s ability to perform at work. That is, negative emotions may motivate negative acts, such as aggression or withdrawal from work tasks, whereas positive emotions may motivate positive acts, such as OCB behaviors [9]. However, PC fulfilment may also improve mental health in a manner similar to its potential relationship with engagement [30], whereby high fulfilment of the PC reflects increased employee resources to cope with stressful situations at work and low fulfilment reflects low resources leading to poorer coping at work and higher mental distress.

### 1.2. Negative Affectivity

In addition to the aforementioned variables, it is important to consider others that may potentially impact the PC and its outcomes. That is, the PC is fundamentally perceptual, and therefore individual differences in personality and personal resources may influence such perceptions. For instance, negative affectivity (NA) may play a role, with high NA having been strongly associated with a harsher appraisal of breach severity and consequently, negative outcomes of the breach, such as poor performance, are more likely for employees with high NA [22,27].

Individuals high in NA have a pervasive, negative view of themselves and the world in general, which may affect their appraisal of situations [46]. Due to the perceptual nature of the PC, and the relationship between feelings of positive affect and high self-worth, individual differences in negative affectivity could affect its relationships with employee outcomes through the appraisal process and sensitivity to a breach, and specifically may influence employee engagement by reflecting less personal resources available due to the individuals’ negative emotionality and ability to build resources needed to be engaged. Further, employees lower in NA might be more inclined to proactively build resources at work and increase job challenges to maintain their engagement [47]. NA may also negatively influence job satisfaction, considering both constructs have a strong evaluative aspect, and positively influence psychological distress because of the negative mental state characteristics associated with both concepts. Thus, researchers have called for the inclusion of NA in studies of the PC [22]. Thus the current study accounts for NA in the relationships between PC, performance, and potential mediators while investigating NA as an important variable in its own right.

The current study aims to contribute to the knowledge of the PC by examining the relatively novel concept of PC status in conjunction with PC breach to predict task performance (IRB) and performance that is beyond formal roles (OCBO and OCBI) while accounting for negative affectivity. Engagement, job satisfaction, and psychological distress will be examined as potential mediators in the relationships between the PC variables and performance. Specifically, positive PC status and negative PC breach were expected to predict higher levels of engagement, and in turn, higher engagement may lead to better employee performance. The PC variables could also influence job satisfaction and distress and that job satisfaction and distress may also affect the two types of OCB, as well as IRB.

## 2. Materials and Methods

### 2.1. Participants

The entire nursing staff of the hospital was invited to participate in the survey. Staff on leave for the survey period were excluded. The survey was paper because of concerns that not all staff may have had access to private web browsers and it was felt by the researchers and the steering committee of staff representatives that staff may prefer paper surveys. The researchers generated the individually-named sealed envelopes containing the survey and the pre-paid reply envelope was addressed to the researchers. The survey was anonymous and voluntary. Sealed, addressed envelopes containing the survey kit were distributed directly to staff. Anonymity was further strengthened by using reply-paid envelopes for the return of the surveys that went directly to the researchers and not via the internal mail system.

### 2.2. Measures

PC status was calculated from measures of PC obligations, fulfilment, and importance. This measure was based on work by Rousseau [48] and adapted from calculations of PC status from two papers [22,31]. A weighted PC status variable was calculated by subtracting the extent that an item was fulfilled from the extent it was obligated, and this term was multiplied by its importance. These scores were then added together for each ‘item’ (e.g., (obligations − fulfilment) × importance of each ‘item’). Each score was then summed to make the final status variable.

Perceived PC breach was measured using a previously-validated five item scale [49]. This scale was measured on a 5-point scale (1 = *strongly disagree* to 5 = *strongly agree*). Example items include “almost all promises made by my employer when I started have been kept so far” and “so far my employer has done an excellent job of fulfilling its promises to me”.

Negative affectivity was measured using a short 5-item version of the PANAS, which has demonstrated good reliability [46,50]. Participants were asked to rate the frequency with which they experienced each item over the past week on a 5-point scale (1 = *very slightly or not at all* to 5 = *very much*). Items included “distressed” and “nervous”.

The employee work engagement scale used 12 items [51] based on Kahn’s three components of engagement [52]. For each item, participants indicated the extent to which they agreed from 1, *strongly disagree,* to 5, *strongly agree*, and included “performing my job is so absorbing that I forget about everything else”, “I get excited when I perform well on my job” and “I stay until the job is done”.

Psychological distress was measured using the K-6 [53] which was adapted from the K-10 developed by Kessler and Mroczek [54]. Participants indicated whether, in the last 30 days, each issue related to their psychological health on a 5-point rating (1 = *all of the time* to 5 = *none of the time*). Examples of items include “did you feel nervous?” and “did you feel that everything was an effort?”

Job satisfaction. The short version of Brayfield and Rothe’s scale [55] as found in Agho, Price, and Mueller [56] was used to measure job satisfaction. Participants rated the degree that they agreed with several statements relating to their job satisfaction, rated from 1, *strongly disagree,* to 5, *strongly agree*. Items included “I find real enjoyment in my job” and “most days I am enthusiastic about my job”.

OCB was measured on the commonly-used OCBO, OCBI, and IRB scales of Williams and Anderson [57]. The discriminant structure and validity of the items has been tested several times including with nurses [58]. All items were scored on a seven-point rating (1 = *strongly disagree* to 7 = *strongly agree*). Example OCBI items included “I help others who have been absent” and “I go out of my way to help new employees”. Example OCBO items included “My attendance at work is above the norm” and “I take undeserved work breaks”. Example IRB items included “I adequately complete my assigned duties” and “I perform the tasks expected of me”.

Structural equation modelling (SEM) was conducted with maximum-likelihood estimation in AMOS [59]. Goodness of fit measures such as a small χ^2^ and non-significant *p* value, using a Bollen-Stein bootstrapped *p* across 2000 samples were used (per [60]). Other standard indices of model fit such as the standardized root mean square residual (SRMR), root mean square error of approximation (RMSEA), and comparative fit index (CFI) were also used (per [60,61]). The dataset was checked for missing values, outliers, and normality. Cases missing more than a third of items in any subscale were removed from the dataset, leaving 177 cases. Further analyses revealed that any further missing data were missing completely at random. Consequently, the missing data were replaced using maximum likelihood estimation.

## 3. Results

A sample of 177 nurses and midwives from a medium to large hospital in Australia participated in the current study, representing a response rate of 39.3%. The respondents comprised 42.5% nursing staff and 57.5% midwifery staff, and 99.5% were female. The majority of respondents worked part-time (64.3%) and had been working in their current position for 1–9 years (55%). The means, standard deviations, reliability coefficients, and correlations of and among the variables are shown in Table 1.

A manifest variable was created for PC status due to the way it was calculated. The measurement model was then checked through a process of cumulative congeneric confirmatory factor analyses conducted on the remaining non-manifest variables. 

Then, for the non-manifest variables, item parceling was used. Item parceling forms aggregate manifest variables and is recommended in SEM by several researchers due to improved psychometric properties [62]. Item parceling gives more stable parameter estimates [63]. The method of parceling used was single indicator latent variables (SILVs), as recommended in other studies (e.g., [64,65]), and work equally as well as other methods [66]. Further, SILVs produce better estimates than summed manifest variables [66]. The formula for creating the SILVs is in [67].

The earlier cumulative congeneric testing of the measurement model also showed that the items had appropriate unidimensionality for the parceled variables and provided support for, admittedly sample-specific, discriminant, and convergent validity, especially by establishing discrimination of each variable from each other variable (per [68]). The full SEM had relationships from the predictor variables (PC status, PC breach, and NA) to the attitudinal and mental health outcomes (psychological engagement, job satisfaction, psychological distress), and those outcomes onto aspects of performance behaviors (OCB and IRB). The initial SEM had χ^2^(14) = 69.99, Bollen-Stein *p* < 0.001. Removing the non-significant paths led to a model with a χ^2^(24) = 104.95, Bollen-Stein *p* < 0.001. As a final check, modification indices suggested a relationship between engagement and job satisfaction be added, leading to a model with χ^2^(20) =24.42, *p* = 0.224, Bollen-Stein *p* = 0.875, demonstrating the model was significantly improved. The final model has a good range of fit indices: χ^2^/df = 1.221, SRMR (0.045), RMSEA (0.035), GFI (0.971) and CFI (0.988).

**Figure 1:**
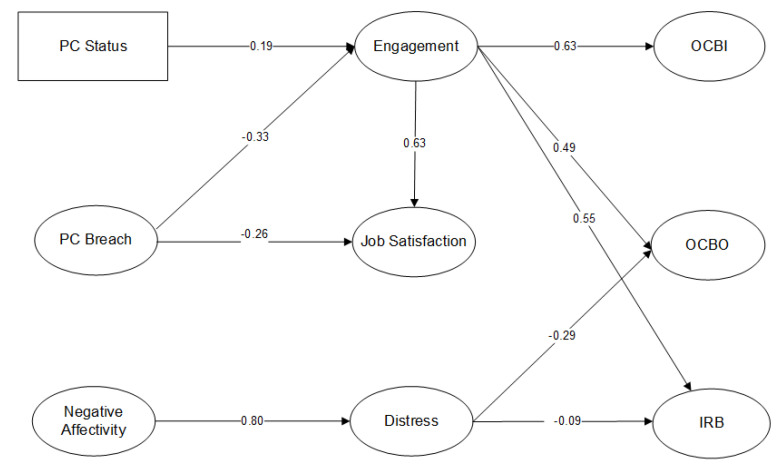
The structural equation model of the significant relationships among the aspects of the psychological contract, via mediators, onto extra-role and in-role performance behaviors.

The final model Is illustrated in Figure 1. In the final model, PC status is positively related to engagement (β = 0.19). PC breach negatively linked to engagement (β = −0.33), while positively linking to job satisfaction (β = 0.26). Negative affectivity positively related to distress (β = 0.80). Engagement related to job satisfaction positively (β = 0.63), OCBI (β = 0.63), OCBO (β = 0.49) and IRB (β = 0.55). Psychological distress was negatively related to OCBO (β = −0.29) and IRB (β = −0.09). 

The final model explained a reasonably high proportion of each of the target variables (OCBO = 34%, IRB = 31%, OCBI = 40%). These high proportions of variance explained in the target variables suggest large effect sizes and that the sample had ample power to detect those large effect sizes. No correlations were allowed between error terms and the correlations between the exogenous variables were 0.33 between PC Status and Breach, 0.10 between PC Status and NA, and 0.24 between NA and PC Breach.

## 4. Discussion

The current study examined the impact of PC status, PC breach, and NA on three performance indicators (i.e., OCBI, OCBO, and IRB) via potential mediating effects of employee engagement, job satisfaction, and psychological distress among nurses in a hospital setting. The results extend previous findings with PC status, representing a novel theoretical contribution to the PC literature. The findings also emphasize the key mediating role of engagement between aspects of the PC and performance, focusing on three types of performance that are not often tested together [42]. Further, the findings demonstrate that psychological distress may mediate the relationship between negative affectivity and organization-focused performance.

### 4.1. PC Status and PC Breach as Distinct Concepts

The current study illustrates that PC status and PC breach are distinct, are differentially related to various outcomes, and may influence employees in different ways. Specifically, PC status and breach were related to engagement, yet only breach was related to job satisfaction, reflecting potentially different mechanisms underlying the impact of the PC status and breach on employees. For instance, high PC status may reflect employees’ expectations of future reciprocation from the employer and the belief that their employers will respond in kind, which may lead to increased motivation represented by higher engagement, leading to higher performance [13,23,35]. In contrast, a PC breach may represent a particular situation where the PC has been damaged, negatively impacting employees’ emotions, such as indicated by mental health and job satisfaction [6,29,30,31], thus lowering their engagement and consequently their performance.

Essentially, the impact of high PC status may at least partially represent the long-term effects of ongoing high PC fulfilment for important obligations, and may therefore be a way to examine the long-term impact of PC on employees. In contrast, a PC breach may represent a more immediate PC event that damages the employees’ belief that their efforts will be reciprocated, while also being related to feelings of betrayal regarding the organizations’ reneging on their promises [27]. Both high PC breach and low PC status may also reflect a scarcity of resources for the employee provided to the employer, presenting a barrier to employee performance.

Generally, the current study supports the need to examine PC on to IRB and OCB simultaneously and highlights the essential role that employee engagement plays in these relationships. As expected, PC status and breach influenced engagement, and engagement subsequently positively related to IRB, OCBI, and OCBO. These findings support previous research indicating that high engagement may predict extra-role and in-role performance [11,13,40,41,43]. To the best of our knowledge, only one study examines a model in which the PC predicts engagement, and engagement subsequently predicts employee outcomes despite researchers referring to engagement as an important consideration for the employee-employer exchange relationship [19,69]. PC status may positively impact engagement through reciprocation processes in the employee-employer relationship [8,36] or through resources related to the fulfilment of the PC.

High PC fulfilment reflects more resources being given to the employee that then enable the employee to engage and perform at work. PC breach was also identified as an important antecedent of low engagement, which may be due to employees’ perceiving the breach as depriving them of the resources that would enable them to be more engaged and motivated to perform [16]. That is, high PC status and low PC breach may represent more resources for employees that enable higher engagement with work tasks [19]. Moreover, this resource mechanism is likely to be cyclic, whereby employees with higher engagement may invest more personal resources into their work and go beyond their role to help the organization and their coworkers [40]. Resources derived from the PC would be expected to enable engagement, which subsequently enables employees to readily identify and build resources, enabling higher engagement and performance [45,47].

Alternatively, reciprocation processes may be responsible for the impact of the PC on employees, and overall the current study supports the influence of social exchange processes on engagement and extends previous research (e.g., [8]) to the PC. Employees may feel obligated to reciprocate the fulfilment of their PC from the organization by increasing their own engagement at work or withdrawing their engagement in response to a PC breach and/or low PC status [70]. PC status provides insight into the role that fulfilment and importance of obligations play in compelling employees to become more engaged, and subsequently, their extra efforts and resources used to reciprocate over-fulfilment of the PC from the employer and conversely withdraw inputs when the organization fails to deliver on promised inducements. That is, over-fulfilment of the PC may increase OCB either by compelling the employee to reciprocate beyond what is formally required of their role to balance the exchange relationship and/or because over-fulfilment may increase the belief that the employer will continue to reciprocate in the future [44]. Consequently, high PC status may also act as a motivator that sets up the perceived need for employees to reciprocate over-fulfilment of obligations by going above and beyond their role (i.e., demonstrating OCBO and OCBI) in addition to maintaining in-role performance (IRB). On the other hand, a PC breach may signal that the organization is unable or unwilling to keep its promises thus decreasing the nurses’ motivation to engage and perform at work.

### 4.2. Mediating Roles of Job Satisfaction and Psychological Distress

Similar to previous research, job satisfaction was negatively predicted by PC breach [6], however, job satisfaction was not related to the employee performance behavior outcomes, in contrast to previous findings that it relates to OCBs [7,15]. Job satisfaction was positively predicted by engagement, which may demonstrate that engagement at work directly makes employees happy in their work. The link between PC breach and job satisfaction may simply be because job satisfaction represents an evaluation or perception of what the employee wants from the job and what they receive, and a PC breach means that the employee is not likely to be receiving what they want [6].

Moreover, the findings of the current study indicate that the relationship between negative affectivity and IRB or OCBO may be mediated by psychological distress, aligning with previous research suggesting that negative emotions decrease employee performance and motivate negative acts, such as withdrawal from work tasks [9]. It also makes intuitive sense that pervasive negative emotionality (i.e., NA) would strongly predict psychological distress, itself a reflection of negative emotionality. That is, employees with high NA are particularly vulnerable to psychological distress, and in this case nurses with high distress may cope by psychologically withdrawing from their work (e.g., lowering their engagement), thereby lowering IRB, and their decreased resources may make these nurses less able or less willing to demonstrate OCBO. Further, the slightly different relationships regarding distress for OCBO and OCBI (where individual-focused OCB was not significantly related to distress) support calls for including the two OCB variables separately in research [42] and help to extend earlier work showing relationships between engagement, distress and overall work performance [14].

However, the absence of a significant relationship between NA and engagement does not align with previous research finding an influence of dispositional characteristics on engagement [8,71], though their previous findings may be based more on positive affectivity increasing engagement and performance rather than NA decreasing these aspects [71]. Some researchers suggest that NA and positive affectivity are opposites on the same spectrum (and therefore fundamentally the same concept; [72]), they are generally treated as being separate and distinct [46,73]. Therefore, future studies could examine both types of affectivity.

The main limitation of the study was its cross-sectional design, and therefore definitive conclusions about causality cannot be determined. Therefore, future studies in this area would benefit from longitudinal data to better investigate causal connections between the variables. Additionally, the current study’s participants were from nursing/midwifery in one organization in a hospital in Australia, and therefore future studies should examine the relationships between the variables across occupations (and/or countries) to check the generalizability of findings. The current study also relies on self report measures, though these measures are appropriate because our theory basis focuses on the perceptions of the PC.

## 5. Conclusions

The quality of the social exchange relationship between employers and nurses represented by the PC is vital for nurses’ motivation, job satisfaction, and mental well-being, which in turn impact nurses’ in-role and extra-role performance. The relatively uncommon construct of PC status assesses the current, ongoing state of the PC and is, therefore, more responsive and informative for nurse managers, rather than waiting for PC breach, which may be difficult to redress. 

The links between aspects of the PC, employee outcomes, and performance demonstrate several ways that nurse managers could improve nurse performance in their organizations. Notably, the associations highlight the importance of improving the fulfilment of the PC, especially for obligations that are important to a particular nurse, and avoiding a PC breach. To address these aspects of the employment relationship, nurse managers should refrain from making promises that they do not reasonably expect to keep and should be careful when setting up expectations of obligations (directly and indirectly), especially during recruitment and ongoing social interactions at work. If promises are made, employers should make every effort to consistently fulfil them or explain the reasons for non-fulfilment if something occurred beyond the employer’s control. Nurse managers should also take care of nurses with potentially high NA, starting with recruitment, so that greater support can be provided for these employees that are at risk of psychological distress and poor performance.

Importantly, the key role that employee engagement plays in nurses’ in-role performance and OCB is strongly demonstrated in the current study. Among nurses, engagement appears to be vital for nurses’ performance. With the current shortage of nurses, OCB and engagement may be more important than ever for this occupational group for maintaining patient satisfaction and quality of care [2,3]. The paper also provides a major contribution to the PC literature, presenting PC status as a novel approach to investigating the PC among employees and demonstrating the importance of exploring PC status in addition to PC breaches in the same study given evidence that they impact employees through different mechanisms.

## Figures and Tables

**Table 1:** The descriptive statistics for all of the predictor and outcome variables later used in the SEM ^1^.

Variables	Mean	SD	Cronbach Alpha	1	2	3	4	5	6	7	8
1. PC status	9.83	29.61	n/a	-							
2. PC breach	13.02	3.85	0.90	0.31 **							
3. Negative affectivity	5.33	1.77	0.62	0.09	0.20 **						
4. Engagement	20.72	2.67	0.82	0.09	−0.22 **	−0.05					
5. Job satisfaction	22.60	4.70	0.90	−0.16 *	−0.32 **	−0.12	0.41 **				
6. Psychological distress	8.23	2.97	0.84	0.05	0.12	0.59 **	0.01	−0.08			
7. OCBI	42.36	4.06	0.80	0.09	−0.16 *	−0.13	0.51 **	0.24 **	−0.08		
8. OCBO	31.35	3.71	0.74	−0.10	−0.28 **	−0.15 *	0.37 **	0.29 **	−0.25 **	0.41 **	
9. IRB	25.70	2.36	0.94	0.03	−0.19 **	−0.19 *	0.48 **	0.23 **	−0.10	0.61 **	0.56 **

^1^ SD = Standard Deviation, n/a = not applicable, is a manifest variable. * *p* < 0.05. ** *p* < 0.01.

## Data Availability

The data are not publicly available due to privacy reasons.

## References

[1] AIHW Health Workforce. https://www.aihw.gov.au/reports/workforce/health-workforce.

[2] Needleman J., Buerhaus P., Mattke S., Stewart M., Zelevinsky K. (2002). Nurse-Staffing Levels and the Quality of Care in Hospitals. N. Engl. J. Med..

[3] Vahey D.C., Aiken L.H., Sloane D.M., Clarke S.P., Vargas D. (2004). Nurse Burnout and Patient Satisfaction. Med. Care.

[4] Organ D.W., Podsakoff P.M., MacKenzie S.B. (2005). Organizational Citizenship Behavior: Its Nature, Antecedents, and Consequences.

[5] Purvis L.J., Cropley M. (2003). The Psychological Contracts of National Health Service Nurses. J. Nurs. Manag..

[6] Zhao H.A.O., Wayne S.J., Glibkowski B.C., Bravo J. (2007). The Impact of Psychological Contract Breach on Work-Related Outcomes: A Meta-Analysis. Pers. Psychol..

[7] Mohammad J., Quoquab Habib F., Alias M.A. (2011). Job Satisfaction and Organizational Citizenship Behaviour: An Empirical Study at Higher Learning Institutions. Asian Acad. Manag. J..

[8] Saks A.M. (2006). Antecedents and Consequences of Employee Engagement. J. Manag. Psychol..

[9] Spector P.E., Fox S. (2002). An Emotion-Centered Model of Voluntary Work Behavior: Some Parallels between Counterproductive Work Behavior and Organizational Citizenship Behavior. Hum. Resour. Manag. Rev..

[10] Du Y., Liu H. (2020). Analysis of the Influence of Psychological Contract on Employee Safety Behaviors against COVID-19. Int. J. Environ. Res. Public. Health.

[11] An M., Shin E.S., Choi M.Y., Lee Y., Hwang Y.Y., Kim M. (2020). Positive Psychological Capital Mediates the Association between Burnout and Nursing Performance Outcomes among Hospital Nurses. Int. J. Environ. Res. Public. Health.

[12] Rousseau D.M. (1989). Psychological and Implied Contracts in Organizations. Empl. Responsib. Rights J..

[13] Chen Y.-S., Lien C.-M., Lo W.-Y., Tsay F.-S. (2021). Sustainability of Positive Psychological Status in the Workplace: The Influence of Organizational Psychological Ownership and Psychological Capital on Police Officers’ Behavior. Sustainability.

[14] Lu X., Yu H., Shan B. (2022). Relationship between Employee Mental Health and Job Performance: Mediation Role of Innovative Behavior and Work Engagement. Int. J. Environ. Res. Public. Health.

[15] Casu G., Mariani M.G., Chiesa R., Guglielmi D., Gremigni P. (2021). The Role of Organizational Citizenship Behavior and Gender between Job Satisfaction and Task Performance. Int. J. Environ. Res. Public. Health.

[16] Chambel M.J., Oliveira-Cruz F. (2010). Breach of Psychological Contract and the Development of Burnout and Engagement: A Longitudinal Study among Soldiers on a Peacekeeping Mission. Mil. Psychol..

[17] Yeh Y.-J.Y., Ko J.-J.R., Chang Y.-S., Chen C.-H.V. (2007). Job Stress and Work Attitudes between Temporary and Permanently Employed Nurses. Stress Health J. Int. Soc. Investig. Stress.

[18] Uchendu C., Windle R., Blake H. (2020). Perceived Facilitators and Barriers to Nigerian Nurses’ Engagement in Health Promoting Behaviors: A Socio-Ecological Model Approach. Int. J. Environ. Res. Public. Health.

[19] Parzefall M.-R., Hakanen J. (2010). Psychological Contract and Its Motivational and Health-Enhancing Properties. J. Manag. Psychol..

[20] Robbins J.M., Ford M.T., Tetrick L.E. (2012). Perceived Unfairness and Employee Health: A Meta-Analytic Integration. J. Appl. Psychol..

[21] Conway N., Briner R.B. (2009). Fifty Years of Psychological Contract Research: What Do We Know and What Are the Main Challenges. Int. Rev. Ind. Organ. Psychol..

[22] Turnley W.H., Feldman D.C. (1999). The Impact of Psychological Contract Violations on Exit, Voice, Loyalty, and Neglect. Hum. Relat..

[23] Gouldner A.W. (1960). The Norm of Reciprocity: A Preliminary Statement. Am. Sociol. Rev..

[24] Argyris C. (1960). Understanding Organizational Behavior.

[25] Robinson S.L., Rousseau D.M. (1994). Violating the Psychological Contract: Not the Exception but the Norm. J. Organ. Behav..

[26] Wu W.-L., Lee Y.-C. (2020). How Spiritual Leadership Boosts Nurses’ Work Engagement: The Mediating Roles of Calling and Psychological Capital. Int. J. Environ. Res. Public. Health.

[27] Morrison E.W., Robinson S.L. (1997). When Employees Feel Betrayed: A Model of How Psychological Contract Violation Develops. Acad. Manage. Rev..

[28] Blau P. (1964). Power and Exchange in Social Life.

[29] Bal P.M., De Lange A.H., Jansen P.G., Van Der Velde M.E. (2008). Psychological Contract Breach and Job Attitudes: A Meta-Analysis of Age as a Moderator. J. Vocat. Behav..

[30] Conway N., Briner R.B. (2002). Full-Time versus Part-Time Employees: Understanding the Links between Work Status, the Psychological Contract, and Attitudes. J. Vocat. Behav..

[31] Robinson S.L. (1996). Trust and Breach of the Psychological Contract. Adm. Sci. Q..

[32] Gakovic A., Tetrick L.E. (2003). Psychological Contract Breach as a Source of Strain for Employees. J. Bus. Psychol..

[33] Restubog S.L.D., Bordia P., Tang R.L. (2006). Effects of Psychological Contract Breach on Performance of IT Employees: The Mediating Role of Affective Commitment. J. Occup. Organ. Psychol..

[34] Suazo M.M. (2009). The Mediating Role of Psychological Contract Violation on the Relations between Psychological Contract Breach and Work-Related Attitudes and Behaviors. J. Manag. Psychol..

[35] Cropanzano R., Mitchell M.S. (2005). Social Exchange Theory: An Interdisciplinary Review. J. Manag..

[36] Coyle-Shapiro J.A.-M., Kessler I. (2003). The Employment Relationship in the UK Public Sector: A Psychological Contract Perspective. J. Public Adm. Res. Theory.

[37] Lambert L.S., Edwards J.R., Cable D.M. (2003). Breach and Fulfillment of the Psychological Contract: A Comparison of Traditional and Expanded Views. Pers. Psychol..

[38] Conway N., Guest D., Trenberth L. (2011). Testing the Differential Effects of Changes in Psychological Contract Breach and Fulfillment. J. Vocat. Behav..

[39] Irving P.G., Montes S.D. (2009). Met Expectations: The Effects of Expected and Delivered Inducements on Employee Satisfaction. J. Occup. Organ. Psychol..

[40] Rich B.L., Lepine J.A., Crawford E.R. (2010). Job Engagement: Antecedents and Effects on Job Performance. Acad. Manage. J..

[41] Diefendorff J.M., Brown D.J., Kamin A.M., Lord R.G. (2002). Examining the Roles of Job Involvement and Work Centrality in Predicting Organizational Citizenship Behaviors and Job Performance. J. Organ. Behav..

[42] Cohen A., Ben-Tura E., Vashdi D.R. (2012). The Relationship between Social Exchange Variables, OCB, and Performance: What Happens When You Consider Group Characteristics?. Pers. Rev..

[43] Wickramasinghe V., Perera S. (2014). Effects of Perceived Organisation Support, Employee Engagement and Organisation Citizenship Behaviour on Quality Performance. Total Qual. Manag. Bus. Excell..

[44] Coyle-Shapiro J.A.-M. (2002). A Psychological Contract Perspective on Organizational Citizenship Behavior. J. Organ. Behav. Int. J. Ind. Occup. Organ. Psychol. Behav..

[45] Xanthopoulou D., Bakker A.B., Demerouti E., Schaufeli W.B. (2009). Reciprocal Relationships between Job Resources, Personal Resources, and Work Engagement. J. Vocat. Behav..

[46] Watson D., Clark L.A., Tellegen A. (1988). Development and Validation of Brief Measures of Positive and Negative Affect: The PANAS Scales. J. Pers. Soc. Psychol..

[47] Bakker A.B., Tims M., Derks D. (2012). Proactive Personality and Job Performance: The Role of Job Crafting and Work Engagement. Hum. Relat..

[48] Rousseau D.M. (1990). New Hire Perceptions of Their Own and Their Employer’s Obligations: A Study of Psychological Contracts. J. Organ. Behav..

[49] Robinson S.L., Wolfe Morrison E. (2000). The Development of Psychological Contract Breach and Violation: A Longitudinal Study. J. Organ. Behav..

[50] Mackinnon A., Jorm A.F., Christensen H., Korten A.E., Jacomb P.A., Rodgers B. (1999). A Short Form of the Positive and Negative Affect Schedule: Evaluation of Factorial Validity and Invariance across Demographic Variables in a Community Sample. Personal. Individ. Differ..

[51] May D.R., Gilson R.L., Harter L.M. (2004). The Psychological Conditions of Meaningfulness, Safety and Availability and the Engagement of the Human Spirit at Work. J. Occup. Organ. Psychol..

[52] Kahn W.A. (1990). Psychological Conditions of Personal Engagement and Disengagement at Work. Acad. Manage. J..

[53] Kessler R.C., Andrews G., Colpe L.J., Hiripi E., Mroczek D.K., Normand S.-L., Walters E.E., Zaslavsky A.M. (2002). Short Screening Scales to Monitor Population Prevalences and Trends in Non-Specific Psychological Distress. Psychol. Med..

[54] Kessler R., Mroczek D. (1992). An Update of the Development of Mental Health Screening Scales for the US National Health Interview Study.

[55] Brayfield A.H., Rothe H.F. (1951). An Index of Job Satisfaction. J. Appl. Psychol..

[56] Agho A.O., Price J.L., Mueller C.W. (1992). Discriminant Validity of Measures of Job Satisfaction, Positive Affectivity and Negative Affectivity. J. Occup. Organ. Psychol..

[57] Williams L.J., Anderson S.E. (1991). Job Satisfaction and Organizational Commitment as Predictors of Organizational Citizenship and In-Role Behaviors. J. Manag..

[58] Rodwell J., Gulyas A. (2013). The Impact of the Psychological Contract, Justice and Individual Differences: Nurses Take It Personally When Employers Break Promises. J. Adv. Nurs..

[59] Arbuckle J.L. (2010). IBM SPSS Amos 19 User’s Guide.

[60] Byrne B.M. (2013). Structural Equation Modeling with Mplus: Basic Concepts, Applications, and Programming.

[61] Hu L., Bentler P.M. (1999). Cutoff Criteria for Fit Indexes in Covariance Structure Analysis: Conventional Criteria versus New Alternatives. Struct. Equ. Model. Multidiscip. J..

[62] Meade A.W., Kroustalis C.M. (2006). Problems with Item Parceling for Confirmatory Factor Analytic Tests of Measurement Invariance. Organ. Res. Methods.

[63] Bagozzi R.P., Heatherton T.F. (1994). A General Approach to Representing Multifaceted Personality Constructs: Application to State Self-Esteem. Struct. Equ. Model. Multidiscip. J..

[64] Densten I.L. (2005). The Relationship between Visioning Behaviours of Leaders and Follower Burnout. Br. J. Manag..

[65] Hamilton J., Tee S. (2010). The Value-Expectations Relationship: Connecting Customer-Perceived Value with the Expectations of Pharmacy-Offered Services. Inf. Manage..

[66] Sass D.A., Smith P.L. (2006). The Effects of Parceling Unidimensional Scales on Structural Parameter Estimates in Structural Equation Modeling. Struct. Equ. Model..

[67] Munck I.M. (1979). Model Building in Comparative Education: Applications of the LISREL Method to Cross-National Survey Data. Ph.D. Thesis.

[68] Hoyle R.H. (2012). Handbook of Structural Equation Modeling.

[69] McBain R. (2007). The Practice of Engagement: Research into Current Employee Engagement Practice. Strateg. HR Rev..

[70] Turnley W.H., Bolino M.C., Lester S.W., Bloodgood J.M. (2003). The Impact of Psychological Contract Fulfillment on the Performance of In-Role and Organizational Citizenship Behaviors. J. Manag..

[71] Christian M.S., Garza A.S., Slaughter J.E. (2011). Work Engagement: A Quantitative Review and Test of Its Relations with Task and Contextual Performance. Pers. Psychol..

[72] Russell J.A., Carroll J.M. (1999). On the Bipolarity of Positive and Negative Affect. Psychol. Bull..

[73] Kaplan S., Bradley J.C., Luchman J.N., Haynes D. (2009). On the Role of Positive and Negative Affectivity in Job Performance: A Meta-Analytic Investigation. J. Appl. Psychol..

